# The Effect of Social Norms on Residential Insecticide Use

**DOI:** 10.3390/insects15040286

**Published:** 2024-04-18

**Authors:** Moshe Gish

**Affiliations:** School of Environmental Sciences, University of Haifa, Haifa 3103301, Israel; mgish@univ.haifa.ac.il

**Keywords:** dietary taboo, domestic pesticides, entomophobia, extermination, household pesticides, household pests, kosher, non-dietary pesticide exposure

## Abstract

**Simple Summary:**

This study explores how the level of religious observance in Jewish households affects their use of insecticides. The study was motivated by the hypothesis that religiosity increases insecticide use due to insects being a strict taboo in Judaism. By interviewing secular and religious families about their insecticide use and their level of aversion toward cockroaches, the research found that religious participants tended to use more insecticides and had a stronger dislike for cockroaches, despite facing similar levels of exposure to insect pests as non-religious participants. The primary factor driving the intensity of insecticide use was found to be religiosity, with pest exposure also being a significant, but secondary factor. This suggests that religious views categorizing insects as “impure” may encourage the use of insecticides. The findings highlight the importance of understanding cultural attitudes toward insects for devising novel insecticide-reduction initiatives that will be sensitive to the social characteristics that could affect insecticide use in various communities.

**Abstract:**

Insecticide products are widely used in homes around the world, despite concerns about their adverse health effects. Variations in insecticide use levels can stem not only from differences in environmental conditions, but also from societal factors. This study investigates the impact of religiosity on insecticide use in Jewish households, hypothesizing that religious families might use more insecticides because insects are considered taboo in Judaism. Data from interviews with 70 families, examining their insecticide use, exposure to pests, aversion to cockroaches, and other predisposing factors, revealed that despite similar levels of pest exposure, religious families reported higher insecticide use and greater aversion to cockroaches. Multiple linear regression analysis identified religiosity as the primary predictor of insecticide use, followed by pest exposure levels. The elevated insecticide use among religious Jewish families may stem from several factors, with the Jewish categorization of insects as “impure animals” that should be strictly avoided likely playing a crucial role in promoting insecticide use. Understanding how attitudes toward insects influence insecticide use across different societies is crucial for health and environmental authorities to develop novel insecticide-reduction initiatives that will be tailored to the unique social characteristics of various communities.

## 1. Introduction

Household insecticides are widely used to control invertebrates that are common in human dwellings such as cockroaches, spiders, and ants (hereafter collectively referred to as “pests”) [1,2]. Insecticides are the primary method of pest control in homes and are often applied routinely regardless of need [3]. A survey in the United States indicated that 75% of households used at least one insecticide product indoors in the previous year [4]. Studies from around the world have also reported widespread use of residential insecticides, leading to a ubiquitous non-dietary exposure to insecticides that is consistently reflected in the chemical monitoring of exposed surfaces and dust in homes and human biomonitoring studies [5,6,7,8,9,10].

Modern household insecticide products are far less dangerous than their predecessors, due to the low toxicity of the predominant class of active ingredients, pyrethroids [2,11]. Despite being labeled as “green extermination” due to their plant origin and low mammalian toxicity [11], there is growing concern about the adverse health effects of pyrethroids and other adjuvants contained in insecticide products [10,12,13,14]. Consequently, health authorities consider the widespread use of household insecticides a public health concern. To address this, public health authorities and organizations implement regulations [15,16,17] and educational programs aimed at lowering the misuse, overuse, and unwarranted use of household insecticides. However, public education programs, which focus on providing reliable information on pests and pest control, may be limited in their ability to modify people’s behavior.

For a behavior change intervention to be effective, it must alter at least one component of the target behavior. The widely used COM-B model of behavior change [18] is useful for determining what needs to change for an intervention to be effective. According to the COM-B model, three interlinked components are necessary for any behavior to take place: capability (knowledge and skills needed to perform a particular behavior), motivation (cognitive and emotional processes that direct and energize behavior), and opportunity (external factors that facilitate or inhibit behavior). Given that the application of insecticides does not require special capabilities, the interaction between motivation and opportunity should predominantly determine insecticide usage patterns.

Interventions aimed at educating the public by providing information on best practices in insecticide use mainly target people’s motivation. These initiatives also address the “opportunity” component by educating individuals on creating home conditions that reduce the need to use insecticides, such as improving sanitation, sealing entry points, and using alternative integrated pest management techniques. The “opportunity” component, however, includes not only physical external factors (“physical opportunity”) but also social factors (“social opportunity”) such as cultural norms and social cues [18]. While it is plausible that social factors influence insecticide use, to the best of my knowledge, there is currently no evidence of whether and how this actually occurs, which hinders the tailoring of intervention programs to various distinct social contexts.

The use of insecticides varies significantly among households within a society [19] and is likely to also vary across different societies and cultures. Differences in physical opportunities could theoretically provide straightforward explanations for observed variations in insecticide use in different societies. These variations could arise from factors intrinsic to the geographic location of each society, including climatic conditions, local pest populations, municipal services and public sanitation, construction and infrastructure standards, advertising and availability of insecticide products and extermination services, local regulations, and literacy rates. Although there is little evidence on how such factors impact residential insecticide use, their effects are expected to be direct and easily understandable. On the other hand, the role of social opportunity, which involves less-tangible elements, as a factor that could influence insecticide use, is both enigmatic and potentially complex. Understanding the potential effect of social opportunity on insecticide use could allow public health authorities and environmental agencies and organizations to tailor intervention programs to the social attributes of different societies.

Gaining insight into the potential effect of social opportunity might be particularly challenging, as various physical and social opportunities can exert simultaneous effects on human behavior [20,21], making it difficult to isolate specific influences. For example, when comparing societies from different areas, it would be difficult to determine whether variations in insecticide use are generated by differences in the natural prevalence of pest species in these areas, cultural norms that relate to pest control, or a combination of these factors.

This study aimed to isolate and test the effects of social opportunity on insecticide use by controlling for physical opportunity. A good way of doing this would be to compare societies subject to similar physical opportunities but characterized by different social opportunities. The ideal setting for such a study would be a place where different cultural groups live in close proximity under similar environmental conditions, and the main cultural difference between them has a direct relevance to attitudes toward pests.

Therefore, I took advantage of a unique setting in which such conditions exist: in Israel, the Jewish population has a broad spectrum of religious adherence, with 45% defining themselves as secular, 16% as orthodox, 14% as ultra-orthodox, and 25% who do not include themselves in any of these categories [22]. While these groups sometimes live in different neighborhoods or towns, since Israel is so small, there are practically no differences in environmental conditions (in the north and center of the country, where the climate is Mediterranean) or other physical opportunities that could explain any significant variations in insecticide use among these groups.

The Jewish religion imposes strict dietary restrictions on its adherents. Food items which are permitted for consumption according to Jewish law are considered “kosher”. Food that is “non-kosher” is strictly prohibited and to avoid any potential exposure to it, religious Jews invest considerable effort to ensure that their everyday environment is free of “non-kosher” items [23].

With locust as a single exception, invertebrate animals, including insects and other arthropods, are considered “impure” and are hence a strict taboo in Judaism [24]. Religious Jews are therefore required to make sure that their food is completely free of invertebrates that could be seen with the naked eye. This is done on a regular basis by inspecting and sometimes soaking fruits and vegetables prior to cooking or eating [25]. Religious Jews also often prefer buying packaged “insect-free” produce, which is grown under special conditions and then inspected and authorized by dedicated religious institutions.

The taboo on “impure” animals extends beyond diet; religious Jews avoid all contact or sight of such animals and part of the ultra-orthodox Jews do not even expose their children to pictures, drawings, or toys that could resemble “impure” animals, to protect them from mental and spiritual harm [26]. Since there is no ban on insecticides in Judaism, I hypothesized that the extreme taboo on insects translates into a high intolerance of household pests among religious Jews, and consequently, greater usage of insecticides compared to neighboring secular Jews. To test this hypothesis, I surveyed religious and secular Israeli families and compared their levels of insecticide use and other various factors that could have affected it.

## 2. Materials and Methods

### 2.1. Sample Characteristics

The study was carried out in urban areas in northern and central Israel from May to August 2022. A total of 70 Jewish, two-parent, heterosexual families voluntarily participated in the study. Half of the families were secular, while the other half were religious. The spectrum of religiosity of the religious families ranged from orthodox to ultra-orthodox. A family’s level of religiosity was determined using three criteria established by the Israeli Central Bureau of Statistics for gauging religiosity among Jews in Israel [27]: self-identification, the types of educational institutions attended by the family’s children, and the types of authorities overseeing those institutions. Participants were recruited through personal acquaintances and with the assistance of contacts within their communities.

Two female research assistants visited families in their homes, where they tested and interviewed only the mothers [ages 22–65, average age: 46 ± 10.47 (SD)] in a quiet room. The research assistants had to be female, since religious Jewish women are not allowed to be alone in a private place with a man who is not their husband or immediate family member [“prohibition of seclusion” [28]]. The decision to interview only women was derived from the need to ask participants to rate their affective attitudes toward pests, in light of research indicating that men tend to report dishonestly on emotions like fear and disgust, which are often stereotypically associated with weakness or femininity [29,30,31]. Interviewing only women on household insecticide use is unlikely to introduce a significant selection bias, as women are substantially involved in the purchase and application of insecticides in their households [32,33].

### 2.2. Data Collection and Procedure

Initially, participants were informed that the study’s topic was “consumption habits and risk factors in the home environment”. The exact topic of the study was disclosed to the participants only after the completion of the aversion test, which preceded the interview. This was done to minimize demand characteristics that could affect the aversion test [34]. Additionally, it was important not to mention insects in the recruitment process, due to the likely reluctance of ultra-orthodox families to volunteer for a study on “impure” animals.

Each interviewed mother signed an informed consent form before the interview started. The study was approved by the ethics committee of the Faculty of Social Sciences, University of Haifa (042/22). In this study I used the questionnaire and a modified version of the aversion test used by Leibovich-Raveh and Gish [35].

#### 2.2.1. Aversion Test

Unlike the study conducted by Leibovich-Raveh and Gish [35], in which pictures of hands holding cockroaches were presented on a computer screen, in this study printed pictures were placed on cards (a total of 20 different pictures), which were presented to the participant in random order. Each participant was asked to rate the level of unpleasantness of each picture on a scale of 1–10. The average value of the unpleasantness ratings for each participant is hereafter referred to as “*Aversion*”. The use of a computer was avoided since it would be considered inappropriate to bring a computer into the home of an ultra-orthodox Jewish family.

#### 2.2.2. Questionnaire

In short, the questionnaire [35] consisted of three sections. The first section contained questions designed to assess indoor exposure to pests, here referred to as “*Exposure*”. This included subjective evaluations of how often family members encountered pests in their home. I avoided direct methods of measuring pest populations such as sticky-trap monitoring [36] because only the pests or pest-related damage tenants are aware of could influence their decision to use insecticides. Since individuals may not always be aware of the presence of pests, or may mistakenly believe that their homes are infested when they are not [37,38,39], subjective assessments of infestation levels were deemed more relevant to this study than direct pest monitoring.

The second section of the questionnaire featured questions aimed at estimating the level of insecticide use in each household, hereafter referred to as “*Insecticide Use*”. It included inquiries about insecticide applications by household members and professional extermination services, and information on the number and types of insecticide products present in the household at the time of the interview. Products for controlling pet ectoparasites, such as spot-on treatments and on-animal sprays, were excluded from the study as they do not pertain to household pests.

The third section included queries about several characteristics of family members and preferences that might suggest a tendency to use or avoid insecticides, henceforth referred to as “*Tendency*”. Factors considered included a desire to avoid exposure to toxic chemicals, the frequency of engagement with nature, and vegetarianism. The research assistants also collected demographic data and information on the family’s religiosity. The term “*Religion*” will hereafter be used to distinguish between religious and secular families.

#### 2.2.3. Household Density

Given that household density can affect infestation levels and insecticide use [39,40,41], the research assistants inquired about the number of people in the household.

Religious Jewish families, often characterized by their large size [42,43], dedicate considerable time and effort to fostering family bonds, and frequently choose to reside in close proximity to their relatives to facilitate daily support and assistance [44]. Therefore, it was not surprising that some participants (primarily religious) said that the actual number of people regularly present in their homes exceeded the count of their immediate family members. For instance, a 56-year-old religious woman reported that despite having no minor children at home, 28 family members, mainly grandchildren, visit her home nearly every day. Similarly, two 63-year-old religious women described comparable situations with 13 and 10 close relatives, respectively, and a 31-year-old religious woman noted the frequent presence of 10 additional people in her household. Hence, the inquiry was broadened to include not only permanent residents, but also those who regularly visit and spend time in the household. Therefore, the term “household density” hereafter refers to the total number of individuals who are frequently present in the home.

### 2.3. Data Analysis

Following the methods of [35], I created separate index values for *Exposure*, *Insecticide Use*, and *Tendency*, which were based on the answers to the corresponding sections of the questionnaire. In a nutshell, for every question, each possible answer contributed a certain number of points to a total grade (index value) in each interview section. Each index value ranged from 0 (low) to 12 (high). For a detailed explanation on how indices were calculated, see [35].

I examined the relationship between household density and *Insecticide Use* within secular and religious families separately, to avoid a potential Simpson’s paradox, where an apparent correlation between factors in an aggregated dataset reverses or disappears when examined within each subgroup [45]. Since both household density and *Insecticide Use* were notably higher among religious families (see results), overlooking the possible confounding effect of *Religion* could have led to a spurious correlation in the combined dataset.

I used a stepwise multiple linear regression analysis to identify possible predictors of *Insecticide Use* out of the following candidate variables: *Exposure*, *Insecticide Use*, *Tendency*, *Aversion*, and *Religion*. All statistical analyses were performed in JASP [46].

## 3. Results

**Figure 1:**
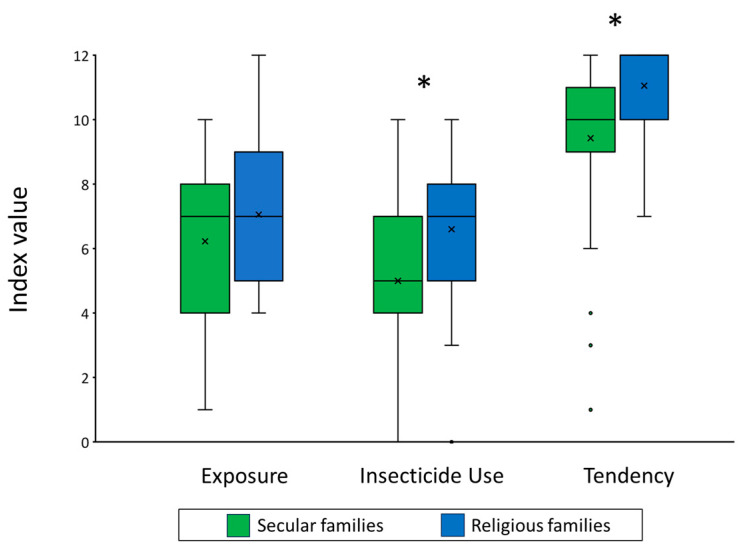
Descriptive statistics for index values used in this study. Asterisks indicate significant differences between secular and religious families. The X mark represents the mean, the line is the median, top and bottom box borders represent the interquartile range (IQR, 25th–75th percentiles), and the whiskers represent minimal and maximal values in the range of 1.5 IQR. Dots represent outliers that exceed 1.5 IQR.

The descriptive statistics for the three index values *Exposure*, *Insecticide Use*, and *Tendency* are presented in Figure 1.

Household density was 4.63 ± 0.27 in secular families and 9.83 ± 0.96 in religious families, and it was not significantly correlated with *Insecticide Use*, neither among secular families (Spearman’s correlation coefficient test; ρ = 0.231, *p* = 0.183), nor among religious families (Spearman’s correlation coefficient test; ρ = 0.295, *p* = 0.085). There was no significant difference in the level of *Exposure* between secular and religious families (6.23 ± 0.39 vs. 7.06 ± 0.38, respectively. Mann–Whitney, W = 495.5, *p* = 0.166). Religious families used more insecticides than secular families (6.6 ± 0.36 vs. 5 ± 0.45, respectively. Mann–Whitney, W = 385.5, *p* = 0.007). The *Tendency* of religious families to use insecticides was higher than that of secular families (11.06 ± 0.22 vs. 9.43 ± 0.44, respectively. Mann–Whitney, W = 338.5, *p* < 0.001).

Before conducting a forward stepwise multiple linear regression analysis, Spearman’s rank-order correlations were calculated to assess potential multicollinearity. None of the pairwise correlations were strong enough to indicate multicollinearity, and therefore I used all factors in the stepwise multiple linear regression (Table 1).

**Figure 2:**
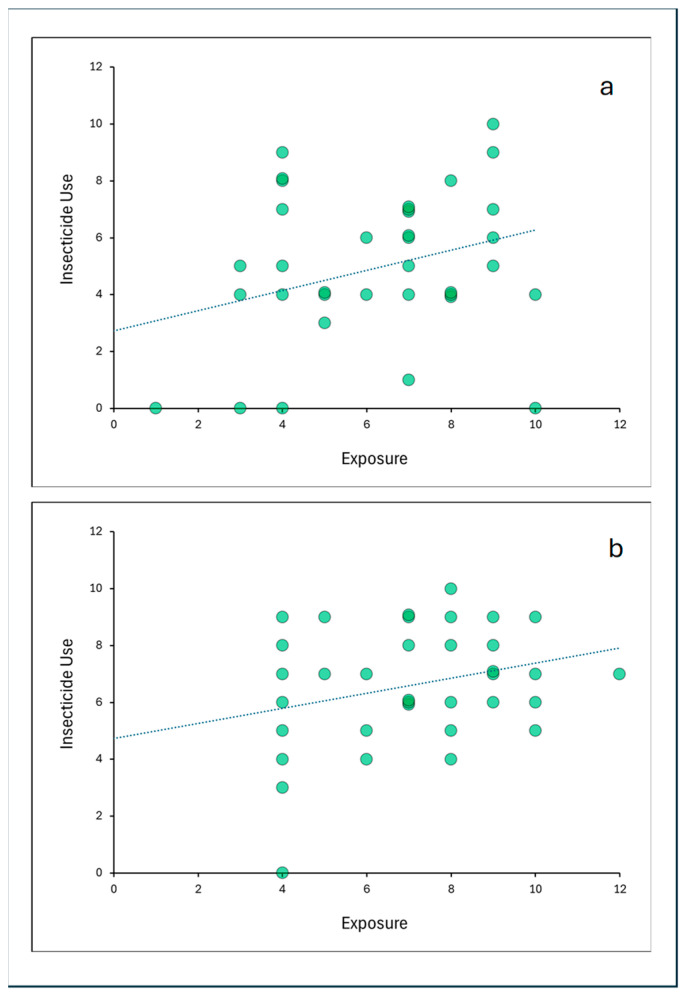
Regression models between *Exposure* and *Insecticide Use* for secular (**a**) and religious (**b**) families. Values on the X and Y axes are index values. The dotted lines are extrapolated regression lines.

I compared the effect of *Exposure* on *Insecticide Use* between secular and religious families (Figure 2) by testing the hypothesis of the equality of the slopes of the two groups using a multiple regression model with an interaction term between *Religion* (dummy variable) and *Exposure* (regressor). The interaction term was not significant (*p* = 0.966), which deems the difference in the slopes insignificant.

**Figure 3:**
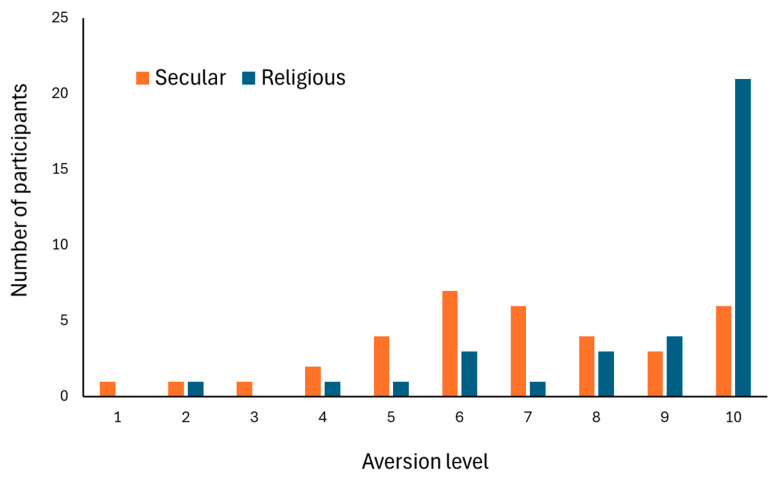
Distribution of the ratings of *Aversion* levels among secular (*n* = 35) and religious (*n* = 35) Jewish women. Women were asked to rate the levels of unpleasantness of 20 pictures of hands holding cockroaches, on a scale of 1–10. A higher score indicates a higher level of *Aversion*. Scores were rounded to the nearest integer.

*Aversion* levels among religious women were higher than those of secular women (mean: 8.64 ± 0.36 median: 10 vs. mean: 6.73 ± 0.4 median: 6.85, respectively. Mann–Whitney, *p* < 0.001). Data distribution was notably different between the two groups (Figure 3).

## 4. Discussion

Religious and secular Jewish families differ in many aspects related to their way of life. One important difference that could potentially be a straightforward explanation of the fact that religious families used more insecticides than secular families is family size, which could affect the frequency of physical opportunities to use insecticides [47]. Following what they believe to be a divine commandment of having many children, religious Jewish families typically have more children than secular Jewish families [42,43]. However, in Israel, the average apartment size of religious and secular Jewish families is similar and therefore household density, defined as apartment size divided by the number of persons living in it [48], is higher among religious families [49]. High household density may be associated with high cockroach infestations [40], likely due to lower sanitation levels that lead to higher infestations [39] and consequently to increased insecticide use [41]. The religious families in this study were indeed larger than the secular families, but family size did not correlate with insecticide use, neither in secular families nor in religious ones. Therefore, I conclude that the higher insecticide use among religious families was not a consequence of higher household density. Moreover, there was no difference between religious and secular families in the level of *Exposure*. Although *Exposure* did affect *Insecticide Use*, this effect was similar in both groups (Figure 2).

The main finding in this study was that *Insecticide Use* was higher in religious families than in secular families despite no apparent differences in the level of *Exposure*. Since religious and secular families also did not differ in the environmental conditions in which they live, housing types, or public sanitation standards, it is likely that the difference in *Insecticide Use* lies in social or personal factors that directly relate to *Religion*, which is overwhelmingly the biggest difference between those two groups. The fact that *Tendency* did not affect *Insecticide Use* means that this index, which comprised several different factors that could potentially affect the family’s inclination to use insecticides, did not indicate a tendency to use insecticides in this study.

An interesting finding that could potentially be linked to *Insecticide Use* was that religious participants had higher *Aversion* levels than secular ones. In the aversion test, participants were asked to quantify their discomfort when exposed to images of cockroaches, using a numerical scale. Since the main component of negative affective attitudes toward insects is disgust [50,51,52,53,54], the ratings given to cockroach pictures likely largely reflected levels of disgust.

Disgust toward insects is a common and intense sentiment in societies where they are not part of the diet [50,53,54,55]. Specifically, a pronounced feeling of disgust toward household pests like cockroaches is considered a normal reaction [54,56,57]. The widespread disgust toward insects is rooted in what is termed “core disgust”, an innate repulsion to potential sources of contamination and harmful stimuli, which helps the individual protect itself against pathogens and maintain bodily integrity [58,59,60,61,62]. Core disgust was likely a significant part of the disgust component of the discomfort expressed by both secular and religious women in this study. However, the heightened *Aversion* reported by religious participants suggests that other forms of disgust, potentially influenced by cultural factors, were at play.

Emotional experiences, including responses to repellent stimuli, are often shaped by one’s cultural background, leading to variations in emotional reactions to similar situations across different societies [63]. The social origins of disgust [64,65] could therefore explain the disparity in *Aversion* levels between secular and religious women. As insects are a religious taboo in Judaism, having insects in the home carries the risk of violating religious law, thus causing a moral transgression that could induce “moral disgust” (disgust elicited by abstract sociomoral transgressions [66]). Religious individuals tend to view harmless violations of sacred norms as moral transgressions of the binding foundations of their society [67] or as violations of spiritual purity, which often elicit feelings of disgust among religious people [68,69,70,71]. This type of disgust as a response to moral transgressions is socially learned and passed from parent to child [72,73]. Moreover, feelings of disgust are sometimes intentionally nurtured and provoked in modern Judaism as a strategy to steer clear of sin [74]. Labeling non-kosher animals as “impure” (the literal meaning of the Hebrew adjective for a non-kosher animal—“tamé”—is filthy/contaminated) serves this purpose, and indeed Jews who adhere to eating only kosher feel disgusted by food that is strictly forbidden to them, such as pork and meat–dairy combinations [75].

Another possible explanation for the elevated *Aversion* among religious women is that, in addition to experiencing discomfort with the images, they may have also used the expression of aversion to underscore their stance on what they perceive as a potential violation of social norms [76]. Additionally, the extreme scores given by religious women may further reflect a desire to convey a positive and virtuous self-image during an interview, as religious individuals might be more inclined to uphold a moral image compared to their non-religious counterparts. This might make them particularly attentive to how they are perceived socially [77,78] and more prone to social desirability in self-report studies [79,80]. Therefore, it is plausible that *Aversion* reflected core disgust among all participants, and in religious participants it also reflected the levels of moral disgust and a desire to emphasize their position on a potential divinity violation while exhibiting socially desirable responses.

Even though *Aversion* was not a predictor of *Insecticide Use* when examined in the linear regression model together with other variables, the fact that it was significantly higher among religious women raises the following question: if *Aversion* is higher among religious participants and also *Insecticide Use* is higher, how come *Aversion* does not contribute to the model predicting *Insecticide Use*? Could it be that decisions to use insecticides are not influenced by the person’s affective attitudes toward the most common household pests? Since this possibility does not seem very likely, it may be that methodological issues were in play [33].

First, the discomfort reported by participants may have reflected not only factors that are likely to affect *Insecticide Use* such as core disgust, but also factors that likely do not affect a person’s inclination to use insecticides, such as social desirability and a wish to display moral purity.

The second issue could lie in the distribution of the data on religious women’s *Aversion* (Figure 3), which indicates an extreme response bias. With 18 out of 35 religious women having an average *Aversion* of 10, and another nine with an average *Aversion* of above nine, data variation may simply be too low for an informative data analysis [33].

The issue of extreme responding in studies asking participants to self-report their affective attitudes toward highly aversive invertebrates (e.g., spiders, cockroaches) is a standing problem in biophobia research [33]. Currently, it limits the ability to gain insight into the causes and effects of extreme biophobia, and therefore the contribution of the *Aversion* rating used in this study to understanding the reasons for the elevated *Insecticide Use* among religious Jews is limited. However, since this phenomenon is likely to have a bearing on human health in religious Jewish communities, it is important to delve into the social norms that facilitate insecticide use in religious Jewish families. This could pave the road for novel intervention plans that will work together with religious leaders in order to mitigate the exposure of their community members to residential insecticides, while encouraging them to consider alternative integrated pest management (IPM) techniques.

This objective may be difficult to achieve with traditional quantitative methods, given the possible homogeneity and limited variability in individual characteristics and reactions in a culture that values conformity and compliance with a comprehensive set of norms encompassing nearly every aspect of everyday life. Therefore, useful insights on the exact reasons for using insecticides among religious Jews, which are the key to effective intervention plans, may be gained most efficiently using qualitative methods and focus groups.

This research offers the first empirical evidence for the influence of cultural factors on the use of insecticides in homes, highlighting the importance of identifying societies prone to excessive or unwarranted use of residential insecticides, and the social norms that drive the problem within those societies. Conversely, understanding how social norms and societal characteristics affect insecticide use may assist in identifying communities that are at risk of a health hazard that could be mitigated through well-informed interventions.

## Figures and Tables

**Table 1:** A forward stepwise multiple linear regression analysis for identifying factors affecting *Insecticide Use* in the homes of secular and religious Jewish families. *Tendency* and *Aversion* were considered but not included in the model.

Explanatory Variable	B	t	*p*
Constant (intercept)	3.323	3.833	<0.001
*Religion*	1.377	2.434	0.018
*Exposure*	0.269	2.171	0.033
Number of observations	70		
R^2^	0.162		
F	6.493		0.003

## Data Availability

The original contributions presented in the study are included in the article; further inquiries can be directed to the author.
